# Effect of Morphology and Structure of MWCNTs on Metal Matrix Nanocomposites

**DOI:** 10.3390/ma13235557

**Published:** 2020-12-06

**Authors:** Íris Carneiro, Sónia Simões

**Affiliations:** 1 DEMM, Department of Metallurgical and Materials Engineering, University of Porto, R. Roberto Frias, 4200-465 Porto, Portugal; up201207199@fe.up.pt; 2 LAETA/INEGI—Institute of Science and Innovation in Mechanical and Industrial Engineering, R. Roberto Frias, 4200-465 Porto, Portugal

**Keywords:** numerical simulation, metal matrix nanocomposites, carbon nanotubes, outer and inner diameter, number of walls, microstructure

## Abstract

The effect of using different carbon nanotubes (CNTs) on the production of nanocomposites was evaluated in this work. The investigated CNTs were multi-walled carbon nanotubes (MWCNTs) with different morphologies and structures. The main objective was to relate the results reported by numerical simulation with the results obtained experimentally in order to validate these methodologies. A detailed characterization of CNTs was carried out to establish the different main characteristics, such as inner and outer diameters, defects, structure and the number of walls. Metal matrix nanocomposites were produced using the powder metallurgy route. The experimental results show that the morphology and structure of MWCNTs have a significant effect on the dispersion process for nanocomposite production. Straight CNTs with a larger diameter and with few defects allow for the production of nanocomposites with uniform dispersion and strong interface bonding, leading to a higher hardness value. In addition, the CNT introduction into a metal matrix induces a change in the deformation behavior that plays an important role in the strengthening mechanisms. Although some aspects are not considered in the molecular dynamic (MD) simulation, such as the CNT random orientation and CNT agglomeration, some comparative relationships can be performed in order to validate some methodologies. While the structure and morphology of the CNTs have a significant influence on the dispersion process, the influence of the diameter and the functionalization treatment on the properties of the nanocomposites is also identified. The experimental results show that the decrease in the diameter of the CNTs and the use of functionalized CNTs also contribute to the obtention of lower mechanical properties of the nanocomposites, as is pointed out in the results of MD carried out in nanocomposites.

## 1. Introduction

Numerical simulation studies of the mechanical properties of carbon nanotubes (CNTs) [1,2,3,4,5,6,7,8,9,10,11] and nanocomposites reinforced with CNTs [12,13,14,15,16,17,18] have attracted significant attention from the research community. Due to the extraordinary properties of CNTs [19,20,21,22,23,24,25,26], such as high stiffness, high strength, high thermal conductivity and low weight, they have a significant number of potential applications. However, there is still difficulty in understanding and determining their properties, which implies a challenge in their application, since conventional characterization techniques cannot be used. In this sense, and to improve the development of components with CNTs, numerical modulation studies help in understanding CNT properties to overcome some challenges observed in experimental work. For instance, in the production of nanocomposites reinforced with CNTs, the mechanical properties are not as high as expected [27,28,29,30,31,32,33,34]. This can be explained by a lack of knowledge of the real properties of CNTs and even their behavior with the metallic matrix during the processing of nanocomposites. Additionally, several factors significantly affect the success of obtaining nanocomposites, such as CNT dispersion, interface bonding between reinforcement and the matrix, volume fraction and the processing techniques. As these variants play an essential role in nanocomposites’ production with the required properties, the numerical simulation of CNTs and its introduction on the metal matrix is extremely useful.

Multi-walled carbon nanotubes (MWCNTs) are the most used as reinforcement material, as they are more economically attractive and easier to manipulate. Nevertheless, most numerical simulation works are based on single-walled carbon nanotubes (SWCNTs), and, thus, the results of most studies regard to the nanocomposites. Therefore, with only a few works related to MWCNTs, to overcome this lack of knowledge, recently, Sakharova et al. [1] reported a study of MWCNT elastic properties under different loading conditions, accompanied by an elaboration of a simplified finite element model. The authors also established a relationship between tensile rigidity and MWCNT characteristics, such as diameters and walls, which allowed them to obtain equations that evaluate the Young’s and shear modulus of MWCNTs with different structures.

In the production of nanocomposites, the homogeneous dispersion of CNTs is crucial to obtain a significant increase in mechanical properties. Chemical functionalization of CNTs is considered to be a good approach to improve the dispersion of reinforcement in metallic matrices. However, this treatment has a significant effect on the structure of CNTs. Singh et al. [35] investigated the effect of chemical functionalization of MWCNTs on the mechanical properties by molecular dynamic (MD) simulation. The results indicate that the MWCNTs functionalized exhibit lower mechanical properties. This can be attributed to the presence of defects due to the functionalization treatment.

There are several methodologies for modeling materials’ mechanical behavior, but the molecular dynamic (MD) approach is the most appropriate to predict the mechanical properties of nanoscale materials. These simulations can be very beneficial in regard to understanding the failure mechanisms at the nanoscale and evaluating the mechanical properties of metal matrix nanocomposites (MMNCs) [1,13].

In some studies, [12,13,14,36,37,38], the mechanical behavior of MMNCs reinforced by CNTs has been studied by molecular dynamics simulations. Silvestre et al. [13] investigated the mechanical behavior of Al-CNT composites under compression by MD simulation. The results showed that the CNTs could be an effective reinforcement of aluminum, providing a potential Young’s modulus increase between 50–100% compared to the pure metal depending on the interface bonding. However, due to the fact that the CNT local buckling results in premature failure, the yield stress and yield strain did not increase. Faria et al. [14] also evaluated the mechanical properties of Cu–CNT composites under tensile and compressive loadings by MD simulations. The study was conducted considering two interface bonding conditions between CNT and Cu that represent the limits that can occur in the production of the nanocomposites. A strong interface bonding results in an increase in tensile and compressive strength, as well as in Young’s modulus. However, a decrease in the yield strain is also observed. The detrimental effect of the CNTs on the yield strain can be explained because of the introduction of the CNTs in the Cu matrix; they induced a different deformation behavior to the Cu without reinforcement. Wang et al. [36] also applied the MD simulation to study the mechanical properties of MMNCs. The effect of chirality, temperature and volume fraction of a CNT-reinforced Ni_3_Al composite was evaluated. Regarding the chirality, the authors noted that the zigzag CNTs can increase Young modulus, while the armchair promotes superior elongation of the composites. The results showed that the CNTs play a critical role in the composites’ deformation behavior since they act as obstacles for the dislocation movement. Increasing the volume fraction induces an increase in the mechanical properties, even at high temperatures. Babu et al. [12] showed the effect of armchair-type CNTs and the temperature in CNTs’ mechanical properties in reinforced nanocrystalline aluminum by MD simulation. In the study conditions, the introduction of the CNTs into a nanocrystalline Al matrix impairs the mechanical properties of the nanocomposites. This detrimental effect of the CNTs can be explained due to the fact that the nanocomposites revealed a higher dislocation density due to the mismatch on the CNT–Al interface. These works show an outstanding contribution to the exploration of what might occur in nanocomposites, providing insight into the influence that CNTs can have on each matrix and respective strengthening mechanisms. The diameter of CNTs also plays an important role in the mechanical properties of nanocomposites. Some authors [37,38] investigated the effect of the diameter of the CNTs on the mechanical properties of the nanocomposites by MD simulation. The results show that with the increase in the diameter of the CNTs, the Young modulus and tensile strength also increase.

While numerical simulations’ importance and convenience in predicting and understanding the behavior or potential of nanocomposites is evident, the association between analytical and experimental results that support them is crucial to validate the used numerical simulation methodologies. However, these numerical simulation studies consider uniform dispersion and apply a load in the CNT axial direction, limiting their validations.

In this context, the present work’s main objective is to understand the effect of MWCNTs with distinct characteristics (such as different diameters, wall number, morphology and functionalization) in the microstructure and properties of metal matrix nanocomposites. Microstructural characterization and hardness tests were conducted to evaluate the effect of the use of MWCNTs with different characteristics on nanocomposite production.

## 2. Materials and Methods

The nickel powders (Goodfellow Cambridge Ltd., Huntingdon, UK) used in the present study were carefully characterized by optical microscopy (OM), scanning electron microscopy (SEM) and even electron backscattered diffraction (EBSD) in previous works [39,40].

The various multi-walled carbon nanotubes used were Nanocyl (Nanocyl S.A., Sambreville, Belgium), Nanothinkx NTX1 and NTX5 (Nanothinx S.A., Patras, Greece) and Fibermax (Fibermax Nanocomposites Ltd., London, UK). The main differences in these CNTs consist of different outer and inner diameters, numbers of walls and functionalization treatment. These different CNTs were chosen in order to evaluate the effect of key features of the morphology and structure of the CNTs on the properties of the nanocomposites and, consequently, the reinforcement of the nanocomposites.

The morphology and structure of the CNTs were characterized by transmission electron microscopy (TEM, FEI Company, Hillsboro, OR, USA) and high-resolution TEM (HRTEM, JEOL Ltd., Tokyo, Japan).

The nanocomposites were produced with 0.75% vol. of CNTs. The mixture and dispersion were ultrasonicated in isopropanol for 15 min, filtered and dried. The powder mixtures were pressed with 900 MPa and subsequently sintered at 950 °C under vacuum for 120 min, based on the conditions reported for the better ones described in previous studies [39,40,41].

The global nanocomposite samples’ microstructural characterization was performed by OM with a Leica DM 4000 M and DFC 420 camera, using the Leica LAS 4.13.0 software (Leica Microsystems, Wetzlar, Germany), where the percentage and size of agglomerations and pores were obtained. Using the OM images, the average grain size was obtained from 200–300 grain measurements per sample, previously chemically attacked to reveal the grain boundaries.

The nanocomposite mechanical characterization was performed by Vickers microhardness tests using Duramin-1 equipment (Duramin-1; Struers A/S, Ballerup, Denmark) with a load of 98 mN, the average microhardness being from 10 to 15 indentations per sample.

For a more in-depth characterization of the produced nanocomposites, the EBSD technique was used to study crystal and microstructural characteristics with high resolution via the analysis of different types of maps and figures using the TSL OIM Analysis 5.2 software (EDAX Inc., Mahwah, NJ, USA). Prior to the map elaboration, the obtained raw data were submitted to a dilatation clean-up, as previously mentioned in other works [40,42], assuming a value of 15° as grain angle tolerance and a minimum of two points for the grain size definition. This step is essential to avoid the wrong indexation of isolated points or a low confidence index, mainly located at grain boundaries, preventing incorrect results and, consequently, conclusions.

In order to evaluate various microstructural characteristics, different EBSD maps were used, and their combination is described. Inverse pole figure (IPF) maps overlapped with the grain boundary contour are useful to characterize the grain size and the crystallographic orientation within each grain and of the global sample simultaneously. The localization of grain boundaries is also possible through image quality (IQ) maps, as they evaluate the quality of the diffraction patterns that are usually weaker on the boundaries due to the overlapping of different patterns. However, this technique is particularly important to highlight more specific microstructural features, such as dislocation cells. The IQ maps are often superimposed with other types of maps in order to highlight and correlate different features, associating the information obtained by the two maps, for example, overlapping IQ maps with IPF maps allows one to associate the misorientation within the grain with possible deformation in the microstructure; furthermore, when high and low angles are delineated in IQ maps, it is possible to relate the formation of dislocation cells (frequently represented by low-angle boundaries) with features present in the microstructure.

## 3. Results

### 3.1. Characterization of the CNTs

**Figure 1:**
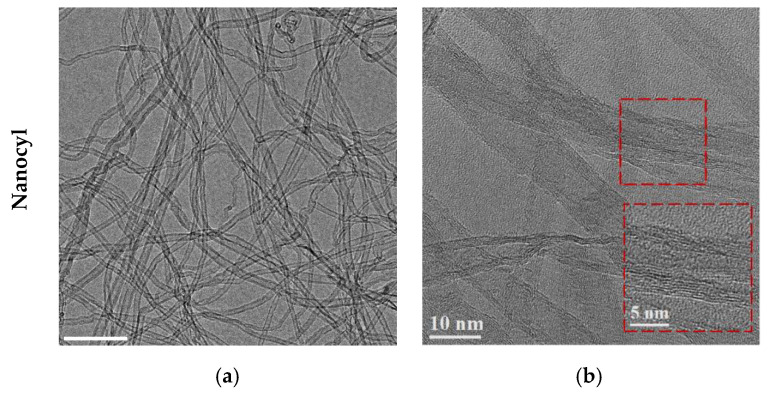

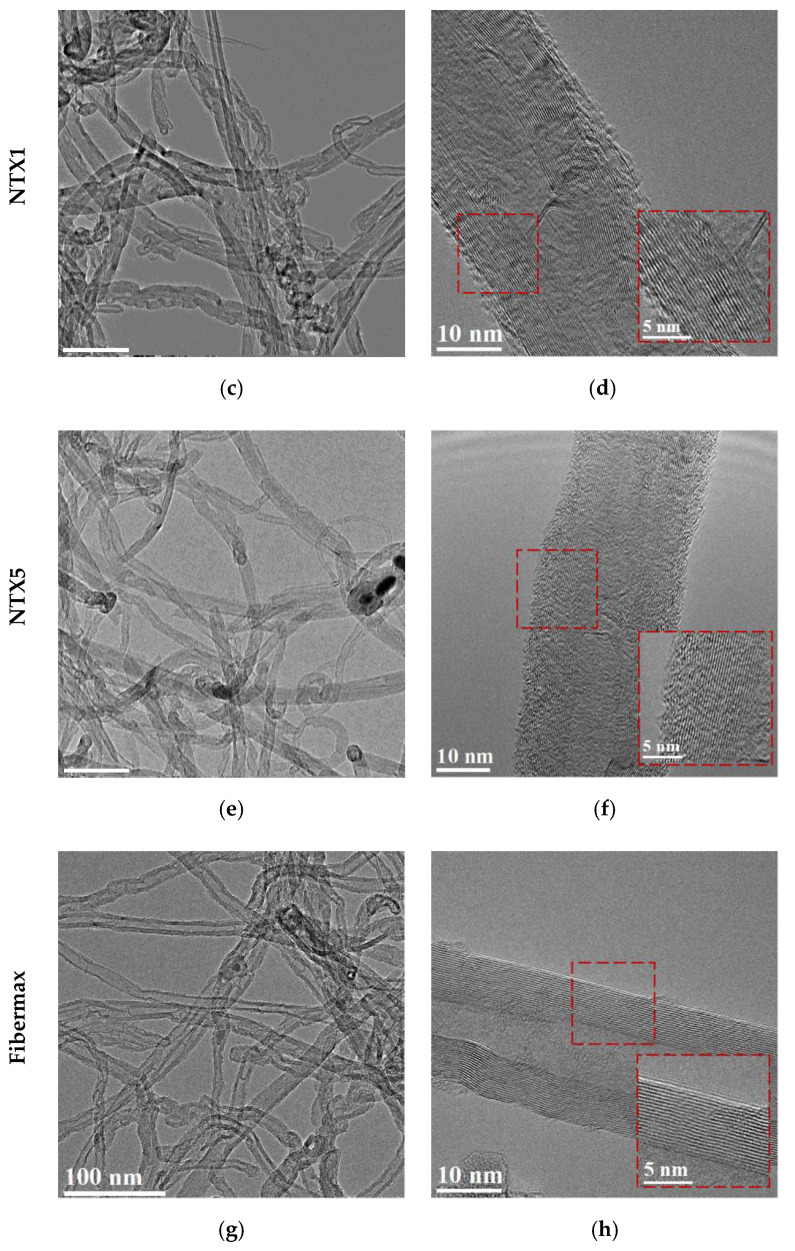
Transmission electron microscopy (TEM) image of the as-received different carbon nanotubes (CNTs) and high-resolution transmission electron microscopy (HRTEM) image showing the morphology and structure of the CNTs for (**a**,**b**) Nanocyl, (**c**,**d**) NTX1, (**e**,**f**), NTX5 and (**g**,**h**) Fibermax CNTs.

In order to characterize the different as-received CNTs that were used, the structure and morphology were evaluated by TEM and HRTEM analysis. The CNTs’ outer and inner diameters and the average number of walls were measured, as shown in Table 1. Figure 1 shows the TEM and HRTEM images of the different CNTs used.

**Figure 2:**
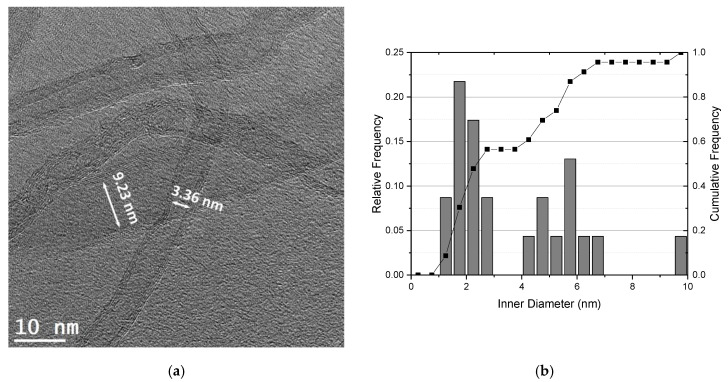
(**a**) HRTEM image of the as-received Nanocyl CNTs showing the presence of CNTs with different diameters and (**b**) inner diameter distribution.

TEM images revealed that the structure and morphology of the CNTs are very distinctive. For instance, the Nanocyl CNTs are those that have a smaller outer diameter and fewer walls. However, despite presenting tubes with the smallest diameter, it is possible to observe some CNTs with larger diameters (about twice the diameter). This variation in diameters of as-received CNTs shows that the diameters*’* separation process after the manufacturing process has not been fully achieved. Figure 2 shows a TEM image and the distribution of the inner diameter of the Nanocyl CNTs, where the presence of tubes with different diameters can be observed. Most CNTs have diameters from 1.2 to 3.0 nm, but CNTs with larger diameters from 4.0 to 9.0 nm can also be observed.

The NTX1, NTX5 and Fibermax CNTs are similar in terms of outer diameter and number of walls. However, the NTX CNTs have more defects in structures, and it is possible to observe the presence of nanoparticles inside them.

**Figure 3:**
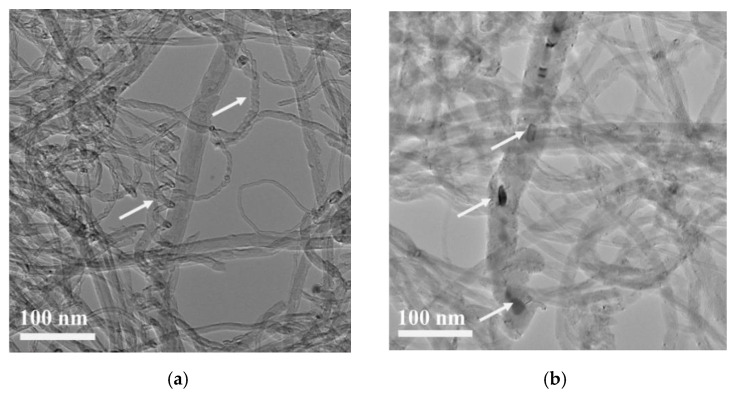

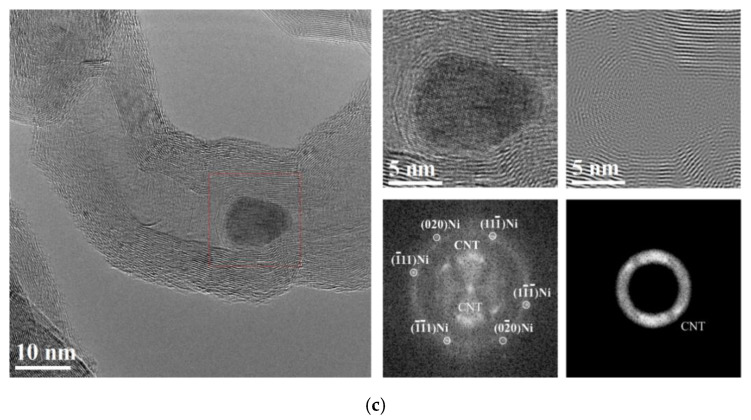
Images of the as-received NTX5 CNTs: (**a**) TEM image showing the CNT deformation (white arrows); (**b**) presence of small second-phase particles inside the carbon nanotubes (white arrows); (**c**) HRTEM images and fast Fourier transform (FFT) confirming the presence of Ni in the CNTs.

TEM images of the NTX CNTs revealed with more detail the deformation and spiral CNT structures in the as-received CNTs. However, some nanoparticles can be observed for the NTX5 CNTs. The same manufacturer produced the NTX CNTs, but the NTX5 are functionalized CNTs. For a more detailed characterization of these NTX5 CNTs, TEM, HRTEM and fast Fourier transform (FFT) analysis were performed (Figure 3). Observing these results, the presence of defects on the CNT structure is clear. This treatment can be important in obtaining a uniform dispersion of CNTs; however, it is necessary to consider that it compromises the mechanical properties, as pointed out by Singh et al. [35]. The nanoparticles present in the CNTs are confirmed as Ni nanoparticles. The presence of Ni, or even Ni_3_C, nanoparticles, is mentioned in the bibliography—both SWCNTs and MWCNTs produced by chemical vapor deposition (CVD) that use Ni-based catalysts [43,44,45,46].

All CNTs exhibited an interlayer spacing ranging from 0.335 to 0.342 nm. This can indicate the type of the CNT, and for this interlayer distance, the armchair type is associated [1,47,48,49]. Numerical simulation studies revealed that the MWCNT structure has an influence on the mechanical properties. For instance, Sakharova et al. [1] concluded by numerical simulation that this armchair carbon nanotube structure presents a slightly higher Young’s and shear moduli compared with zigzag-structured ones, which can be an advantage of using them as reinforcement. In nanocomposite production, the structure of the CNTs can affect their final properties. For instance, Xiang et al. [37], reported that armchair CNTs provides more significant mechanical properties of enhancement to the nanocomposites than the zigzag ones do; in contrast, Wang et al. [36] observed that the zigzag CNTs offer more strength for raising the elastic modulus, while armchair CNTs induces superior elongation of the nanocomposites.

The effects of the number of walls, inner and outer diameters and the presence of a defect on the structure of CNTs in regard to their mechanical properties or even on the improvement in the mechanical properties of the metal matrices are not entirely understood. Although the morphology and structure affect the mechanical properties of CNTs, the success of reinforcement of metallic matrices also depends on other factors, such as a good dispersion of CNTs, a strong interface bonding between the reinforcement and the matrix, and even the possibility of reaction between the matrix and reinforcement. In this sense, it is important to identify and understand how the morphology and structure of CNTs can affect the mechanical properties of nanocomposites, but also to determine whether these characteristics can affect other key factors in the production of these nanocomposites. In order to evaluate and validate the effect of the morphology and structure of CNTs on the production of nanocomposites, different MWCNTs were used as reinforcement materials. The main objective is to demonstrate that it is crucial to predict these CNTs’ properties to use them successfully in the production of MMNCs.

### 3.2. The Influence of Different CNTs on MMNC Production

**Figure 4:**
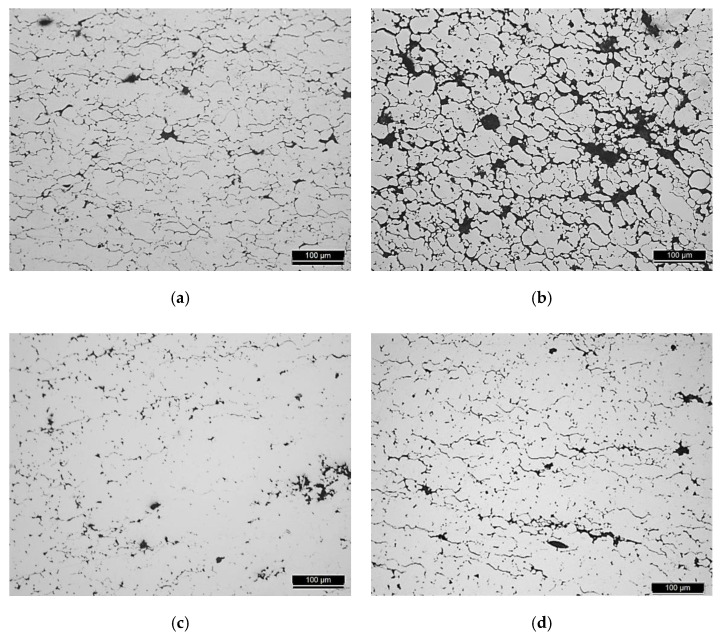
Optical microscopy (OM) images of the nanocomposites produced with the different CNTs: (**a**) Nanocyl, (**b**) NTX1, (**c**) NTX5 and (**d**) Fibermax.

The influence of using different CNTs on the production of Ni/CNT nanocomposites was evaluated by optical microscopy (OM), which was used to characterize the global microstructure of the samples, as shown in Figure 4. It is possible to observe evident differences between the samples, namely in the fraction and size of each phase, with the bright phase corresponding to the Ni matrix and the darker phase corresponding to pores or even clusters of CNTs that are not distinguishable by OM.

**Figure 5:**
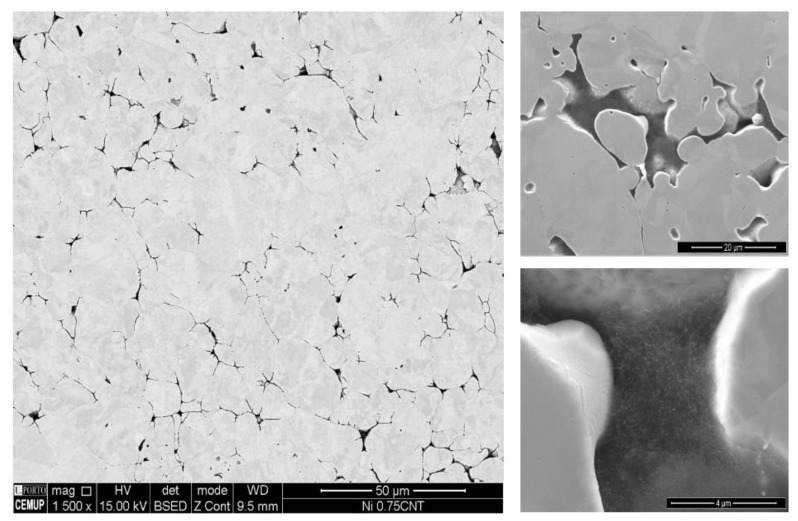
Scanning electron microscopy (SEM) images of the nanocomposite microstructure showing high-magnification images of the pores, confirming the presence of CNT agglomerates.

A scanning electron microscopy (SEM) study was performed in order to confirm the presence of the CNTs in the pores and grain boundaries of the nanocomposites. SEM images presented in Figure 5 shows the microstructure of the Ni/CNT nanocomposite produced with Fibermax CNTs as a representative example. This observation allowed us to understand that at high magnifications, the darker phase observed by OM is indeed associated with CNT agglomerates that are mainly located at pores and grain boundaries.

The OM microstructure characterization of the produced nanocomposites was performed by the measurement of grain size and by determining the fraction (%) and the maximum size of CNT agglomerates and pores. These results are present in Table 2, in addition to the results of microhardness tests.

Based on the results, nanocomposites using Fibermax, Nanocyl and NTX5 CNTs revealed similar results. These nanocomposites exhibit the lowest fraction (%) and size of CNT agglomerates and pores, which means that the dispersion of CNTs was effective. These results are in accordance with OM observations, where a smaller fraction of the darker phase (CNT clusters) is observed for these nanocomposites. The nanocomposite results produced using NTX1 CNTs revealed that the dispersion of the CNTs was not uniform, indicating a higher fraction and a higher maximum size of the CNT agglomerates and pores.

Regarding the average grain size, the sintered samples exhibit similar values. The nanocomposites’ mechanical properties, evaluated by microhardness tests, showed some slight differences. The higher microhardness values were obtained from the nanocomposites produced with NTX5 and Fibermax CNTs. All nanocomposites exhibit an increase in hardness values when compared to the value obtained for Ni produced in the same conditions without reinforcement [40].

Based on the microstructural characterization combined with hardness values, the use of different CNTs significantly affects the dispersion/mixing process and, consequently, the strengthening of the Ni matrix. Higher microhardness values were obtained for the nanocomposites produced with NTX5 and Fibermax CNTs, while the lowest value was observed for NTX1 CNTs. Due to the characteristics of NTX1 CNTs, uniform dispersion was not obtained; this may be because CNTs are very entangled and are characterized by the presence of many defects that make the dispersion more difficult and lead to a weak bonding to the matrix. Functionalization treatment is crucial for NTX CNTs. For nanocomposites produced with NTX5 CNTs, dispersion has previously been successful. Since the functionalization treatment promotes a better dispersion and bonding of CNTs, a more significant increase in the hardness value compared to nanocomposites produced with Fibermax CNTs was expected. However, the hardness values of the nanocomposites produced with NTX5 and Fibermax CNTs are similar. This result can be explained by the presence of many defects and also by the morphology of NTX5 CNTs, which can be detrimental to the obtention of a good bonding between the CNT and the matrix, which promotes a decrease in mechanical properties. The use of shorter and thinner CNTs did not reveal an advantage for the production of these nanocomposites since the results related to the dispersion of the CNTs for the Ni/CNT with Nanocyl CNTs revealed similar values close to the nanocomposites using NTX5 or Fibermax CNTs. However, the hardness value is slightly lower than that obtained for the other nanocomposites. This may be related to the fact that these CNTs are characterized by the presence of tubes with very different diameters. Despite the limitations existing in the comparison between MD and experimental works, it can be observed that the experimental results are in accordance with some of the results obtained by MD, despite the fact that the MD does not consider the presence of clusters and the random orientation of CNTs. For instance, with the increase in the diameter of the CNTs, there is an increase in the hardness of the nanocomposites. Regarding functionalized CNTs, the dispersion obtained is better than expected, and, therefore, the lower hardness value can be justified due to the presence of defects in the structures of these CNTs, which reduced their potential for reinforcement with a decrease in mechanical properties.

In fact, other authors report the influence of morphology and the diameter of CNTs on the dispersion into metallic matrices [50]. Esawi et al. [51] observed that with an increase in MWCNT amount, bent and entangled CNTs with a small diameter were more difficult to disperse than stiff and straight CNTs with a larger diameter. These results can be explained by the fact that smaller CNTs exhibited larger interfacial surface areas per unit volume as well as bent and entangled morphologies. As expected, the strengthening effect of the CNTs is more effective for the nanocomposites produced with the larger CNTs. Dispersion is an essential factor in the reinforcement effect, but the damage of the CNTs that occurs during the process and the reaction between the CNT and the matrix also influence the nanocomposites’ properties. The authors report more significant damage and stronger influence of carbine formation when using CNTs of smaller diameter. Choi et al. [50] also investigated the effect of the structure and morphology of the CNTs (SWCNTs, double-walled CNTs and MWCNTs) in Al–CNT nanocomposites produced by mechanical alloying. Nanocomposites produced using MWCNTs were characterized by a uniform CNT dispersion and with significant improvement in the mechanical properties. Using double-walled CNTs and SWCNTs, the nanocomposites exhibited higher porosity and poorer dispersion.

**Figure 6:**
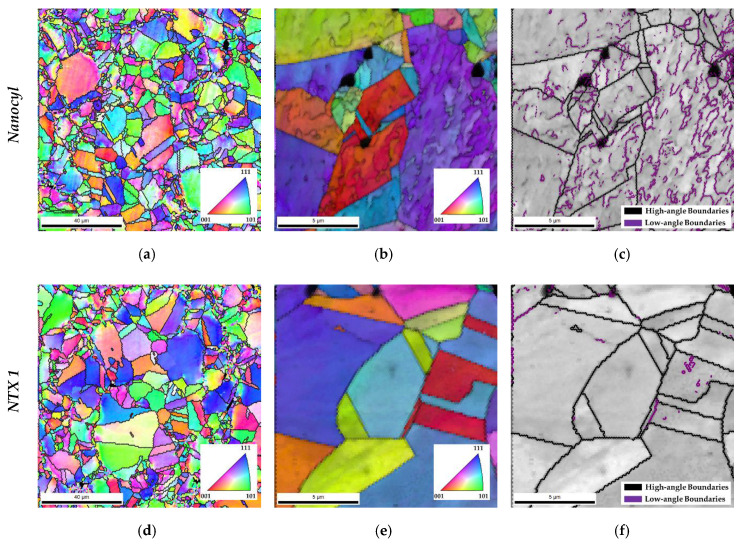

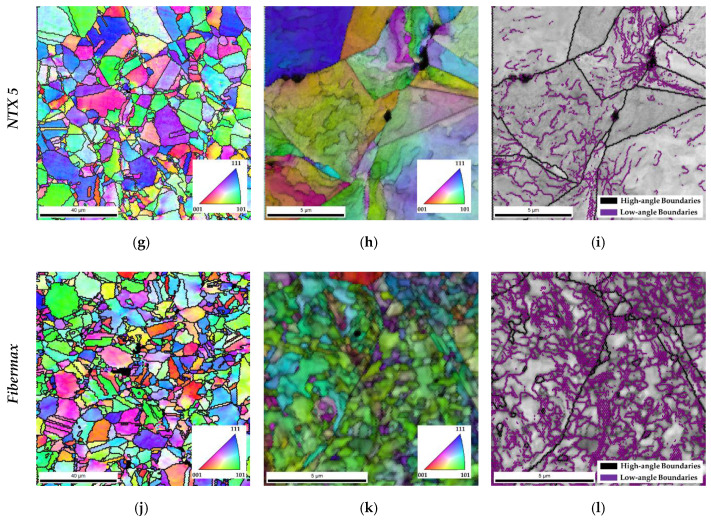
(**a**,**d**,**g**,**j**) Inverse pole figure (IPF); (**b**,**e**,**h**,**k**) IPF superimposed with image quality (IQ); (**c**,**f**,**i**,**l**) IQ with high- and low-angle boundaries delineated, obtained by electron backscattered diffraction (EBSD) of the nanocomposites produced with different CNTs.

An EBSD analysis was performed to evaluate in more detail the influence of different structures and morphologies of the CNTs on the Ni/CNT microstructure after sintering, as shown in Figure 6.

Grain size analysis, misorientation and deformation are essential and are related to the nanocomposites’ mechanical properties. EBSD analysis confirms the effect of the morphology and structure of the CNTs on the dispersion of the reinforcement and, consequently, in the nanocomposite’s microstructure. IPF maps with the delineated grain boundaries show that for the nanocomposites with uniform dispersion (using NTX5 and Fibermax), the grain size is also uniform. For the other nanocomposites, it is clear that close to CNT agglomerates and pores, smaller grains are observed. This is due to the fact that it is more difficult for grain growth to occur during sintering of grains located near CNT agglomerates and pores. The grain size heterogeneity has a significant effect on the nanocomposites’ properties since it presents harder or softer areas depending on the location of the grains. The nanocomposite microstructures also revealed different colors in these IPF maps, meaning that no preferential crystallographic orientation was observed. However, some misorientation within the grains can be observed.

In order to characterize in more detail, the grain misorientation, higher-magnification EBSD maps of the nanocomposites were performed, overlapping IQ with IPF maps and delineating the high- and low-angles boundaries. Nanocomposites produced with Fibermax CNTs exhibited a higher gradient of grain misorientation related to a high fraction of low-angle grain boundaries. The fraction of these grain boundaries is lower for NTX5 and Nanocyl CNTs and almost inexistent for NTX1 CNTs. In previous works [39], a higher fraction of low-angle grain boundaries was observed for nanocomposites when compared with those in the sample of Ni without reinforcement. The deformation mainly originated during the production of the samples, especially in the dispersion and mixing steps, which were eliminated during the sintering of the Ni matrix. However, the CNTs act as obstacles to the dislocation rearrangement of the nanocomposites, hindering the recovery and recrystallization process and resulting in a microstructure with a higher fraction of dislocations. Since a uniform dispersion characterizes the nanocomposites produced using Fibermax CNTs, the effect of the CNTs in the movement of dislocation promotes the formation of grains with a misorientation gradient. In addition, CNT clusters also have a significant effect on the movement of high-angle grain boundaries. A microstructure consisting of grains with different sizes is observed for nanocomposites with a high fraction of CNT agglomerates and pores. This is due to the fact that it is more difficult for grain growth to occur during sintering of grains located near CNT agglomerates and pores.

**Figure 7:**
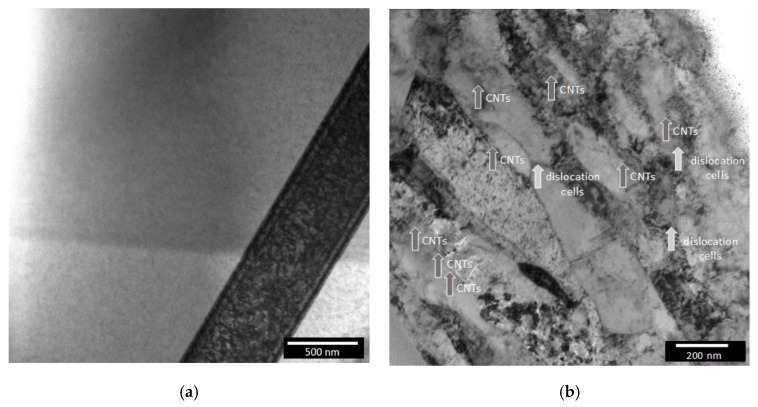
TEM images of (**a**) nickel and (**b**) Ni nanocomposites produced with Fibermax CNTs under the same conditions.

Figure 7 shows the TEM images of the Ni and Ni/CNT nanocomposites produced under the same conditions.

The nanocomposites are characterized by the presence of dislocation cells and individual CNTs well dispersed into the matrix. In addition to the observed clusters, these CNTs inside the Ni grains are essential for effective reinforcement of nanocomposites. Numerical simulation works [13,14,36] in the mechanical properties of MMNCs report that the interface bonding between the CNTs and matrix plays a crucial role in the improvement of the mechanical properties. For instance, Faria et al. [14] showed that a strong interface bonding between CNT and the Cu matrix results in an increase in the tensile and compressive strengths, as well as in Young´s modulus.

Regarding deformation behavior, the effect of the CNTs is evident in the microstructure of the nanocomposites. While the Ni sample without reinforcement is characterized by micrometer grains apparently free of deformation, the nanocomposites exhibit a microstructure with dislocation cells mainly near the presence of CNTs.

**Figure 8:**
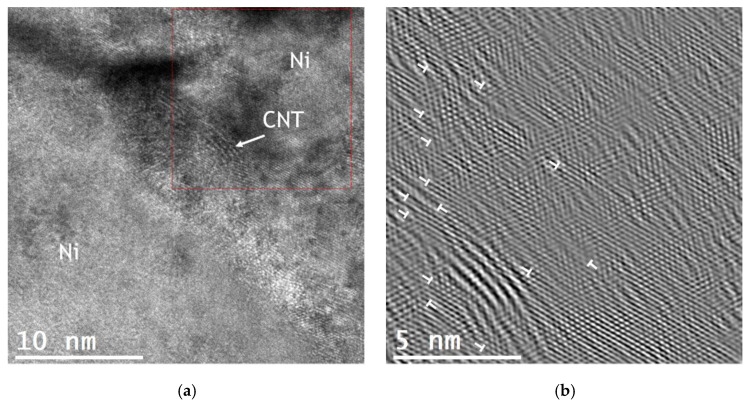
(**a**) HRTEM image showing the Fibermax CNTs in the nickel matrix and (**b**) FFT of the marked zone of (**a**) showing the existence of dislocations.

HRTEM combined with FFT analysis was performed on the nanocomposites to achieve a broader understanding of the CNTs’ influence on the formation of dislocation cells, which is represented by the example of the Ni/CNT produced with Fibermax CNTs in Figure 8.

There are visible lattice deformation and a high number of dislocations around the CNTs. The introduction of the CNTs into Ni matrix significantly affects the microstructure of the nanocomposites. While for the Ni sample, grains without dislocations were observed, the nanocomposites are characterized by the presence of plastic deformation related to the presence of dislocation cells. As already mentioned in previous works [39,41,42], this plastic deformation occurs during the powder metallurgy route that for the nanocomposites is not eliminated in the last step of the process—sintering. During sintering, the dislocation rearrangement did not occur in the same way for the nanocomposites and for the Ni sample without reinforcement. CNTs hinder the movement of dislocations, preventing their rearrangement and annihilation during the recovery and recrystallization processes activated by the sintering temperature.

In fact, the role of the CNTs in the deformation behavior of the metal matrix has previously been appointed by some of the numerical simulation studies [12,14,36], but under compressive and tensile loading. For instance, Wang et al. [36] noted that the CNTs had a significant effect of the deformation regime under tensile strength, acting as obstacles for dislocation rearrangement. Faria et al. [14] also reported the effect of the CNTs on the plastic deformation of the Cu matrix. The nanocomposite revealed a different dislocation pattern to Cu without reinforcement under tensile and compressive loadings. In addition to the load transfer between the matrix and the well-dispersed CNTs, the effect of the reinforcement on the deformation behavior of the matrix also contributes to an increase in the nanocomposite’s mechanical properties.

## 4. Conclusions

In this study, the effect of the morphology and the structure of the MWCNTs on the microstructure and mechanical properties of metal matrix nanocomposites was investigated. The main objective was to validate the results of numerical simulation methodologies used to predict the strengthening effect of the CNTs into a metal matrix. MWCNTs with different diameters, length, number of walls and morphology were used to reinforce the Ni matrix. The nanocomposites were produced by the powder metallurgy route. The structure and morphology of the CNTs have a huge effect on a crucial step in the production and dispersion process. Nanocomposites with enhanced mechanical properties were produced with a larger diameter and straight CNTs, since the dispersion was difficult for bent CNTs with small diameter and the presence of defects. In addition, the introduction of CNTs in the Ni matrix induces a change in the deformation behavior, which has a significant effect on the strengthening mechanisms of the nanocomposites. Despite the limitations of the experimental works and numerical simulations, since the random orientation of CNTs and CNT agglomeration are not considering in the MD works, it was possible to experimentally validate some MD methodologies used in metal matrix nanocomposites reinforced with CNTs. As the diameter of the CNTs increases, there is an increase in the hardness value of the nanocomposites. On the contrary, the functionalization treatment of CNTs promotes the formation of defects in the structure that impair the hardness value obtained for the nanocomposites.

## Figures and Tables

**Table 1:** The average number of walls, outer and inner diameters and length of the carbon nanotubes (CNTs).

CNTs Characteristics	Nanocyl	NTX 1	NTX 5	Fibermax
Number of Walls	6 ± 1	22 ± 9	21 ± 14	17 ± 6
Outer diameter (nm)	8.7 ± 3.0	21.0 ± 7.7	16.4 ± 9.3	18.7 ± 6.5
Inner diameter (nm)	3.6 ± 2.2	5.7 ± 1.5	5.5 ± 0.9	5.0 ± 1.0
Length (µm)	1–3	10	10	1–25

**Table 2:** CNT agglomerate and pore fraction (%) and size, grain size and Vickers microhardness values of the nanocomposites produced with different CNTs.

Nanocomposite Microstructure Features		CNT-Reinforced Ni Matrix			
		Nanocyl	NTX1	NTX5	Fibermax [40]
CNT agglomerates and pores	Fraction (%)	9	16	6	4
	Max. Size (µm)	162	306	155	155
Average Grain Size (µm)		10 ± 5	12 ± 7	9 ± 3	13 ± 5
Microhardness (HV0.01)		136 ± 8	132 ± 7	149 ± 8	155 ± 6
